# The Impact of COVID-19 Pandemic on Stock Market: Evidence from Romania

**DOI:** 10.3390/ijerph18179315

**Published:** 2021-09-03

**Authors:** Mariana Hatmanu, Cristina Cautisanu

**Affiliations:** 1 Faculty of Economics and Business Administration, “Alexandru Ioan Cuza” University of Iași, 700506 Iași, Romania; 2 CERNESIM Environmental Research Center, ICI, “Alexandru Ioan Cuza” University of Iași, 700506 Iași, Romania; cristina.cautisanu@uaic.ro

**Keywords:** COVID-19 pandemic, BET index, economic activity, the ARDL Bound cointegration test, Error Correction Model (ECM), Romania

## Abstract

The current health crisis has several socioeconomic influences that could be compared to those experienced during the 2008 economic and financial crisis. Governments around the world are making great efforts to sustain markets as there are signs showing that the health crisis will be followed by an economic crisis. In this study, we aim to investigate the impact of COVID-19 on the Romanian stock market. For this purpose, we considered the influence on the Bucharest Exchange Trading (BET) index of such variables as the number of new cases and the number of new deaths caused by COVID-19, measures taken by authorities, and the international economic context. The collected data covered the period between 11 March 2020 and 30 April 2021. The Autoregressive Distributed Lag (ARDL) Bound cointegration test was used to measure the impact of COVID-19 on the stock market. The results showed a significant long-term negative impact of the pandemic on the BET index for Romania, while the European economic context had a positive influence. Therefore, these results could be used by authorities as a good guideline for the efficient management of measures that aim to reduce the negative effects of the healthcare crisis.

## 1. Introduction

Globally, humanity has been through several epidemics and pandemics. The SARS-CoV-2 virus led to the latest of the deadly diseases circulating in society. This virus has been viewed as being unique through its high number of symptoms and high transmission rate. This economic crisis caused by the current pandemic differs from the previous ones, such as the Great Depression of the 1930s and the Great Recession of 2007–2009, due to the fact that it involves multiple uncertain socioeconomic links. It was caused in large part by concerns related to the spread of the Coronavirus Disease 2019 (COVID-19) and the governmental policies aimed at limiting person-to-person contact. The health concerns of the public and the stay-at-home and shutdown orders designed to limit contact reduced cash flow to businesses and increased the number of unemployed workers [1].

Governments took unprecedented measures to protect the population’s health and business operations. For example, European states provided significant funds to support companies in difficulty, and additionally, delayed tax payments with no interest or fees for the delays, temporarily cut taxes, implemented measures for the most affected sectors, such as paid leaves due to unemployment, etc. [2].

History has shown that no government can deal with the threats caused by a pandemic or a large-scale healthcare crisis. Therefore, at the World Health Assembly, the 194 members of the WHO adopted on the 31 May 2021 the decision to discuss a new international treaty on pandemics at a special session to be held in November of 2021. Such a treaty would support international efforts to reinforce global health security, in particular on the preparedness and response to health emergencies, in light of lessons learnt from the pandemic [3].

The economic effects of the COVID-19 pandemic appeared in every country under the impact of internal profile related to the spread of coronavirus and the measures adopted for its limitations and amplified by the globalisation and interconnectedness of economies. In most countries, stock markets were negatively influenced by the spread of the COVID-19 disease [4,5,6,7], by the implemented movement restriction policies [8,9,10], and by the uncertainties appearing in the global economy [11,12].

This paper aims to analyse the impact of the COVID-19 pandemic on the Romanian stock market. The analysis of the stock indices is a tool used for describing the evolution of a specific economy. It is a method that calculates the potential of an economy and of unprofitable and low-profit economic activities. To measure the impact of the COVID-19 pandemic on the Romanian stock market, we will be analysing the evolution of the BET index, which is the reference index for the local stock market.

We have set the following objectives: (i) to identify the existence and the type of relations among the studied variables and (ii) to develop an econometric model in order to describe the evolution of the BET index against the analysed influence factors. For this purpose, we will analyse the evolution of the BET index under the influence of the variables reflecting the spread of COVID-19 in Romania (the number of new cases, the number of deaths), as well as of the variables related to the measures adopted by the authorities (restrictions of internal movement, international travel control, monetary policy measures). In addition, the analysis will include the variables describing the international economic context (Purchasing Managers’ Index from Europe, USA, and China, crude oil price). The ARDL Bounds testing approach to cointegration will be used. The data have been analysed over the period from 11 March 2020 to 30 April 2021. During this period, a historical event occurred for the Romanian capital market: it was included into the category of emerging economies. In this context, we decided that it was adequate to analyse the evolution of BET against the factors of influence for the two sub-periods: before and after the inclusion of Bucharest Stock Exchange (BSE) under the category of emerging economies.

Our results show that further in-depth studies are needed for investigating the impact of the COVID-19 pandemic on the stock market and on other socioeconomic activities. Most of the existing literature has studied a group of countries (e.g., East Asian Countries, West African Economic and Monetary Union’s Countries) or regions (e.g., Europe, North America, South Africa), and only a few studies focused on countries that had been severely affected by the COVID-19 pandemic (e.g., China, USA, United Kingdom, Italy). Thus, most of the identified studies have applied panel data methodologies. For a more accurate view on what has been the worldwide impact of the pandemic, more detailed studies are needed considering the socioeconomic specificity of each country. The methods used in such studies include time series and our paper has adopted the same line of research.

Our results are consistent with other similar results reported by the literature in the field and show that the evolution of BET has been affected by movement restrictions and has also been significantly influenced by the monetary policy interest rate reductions, as well as by the economic context in Europe, the USA, and China.

Furthermore, we will present the relevant results found in the literature in the field, the analysed data, the research methodology, and the empirical results. The article also comprises the discussion and conclusions regarding the obtained results.

## 2. Literature Review and Research Hypotheses

With the emergence of the COVID-19 pandemic, the world’s stock markets had to face great uncertainties [8,11,12]. Thus, most stock market indices around the world have registered their biggest one-day falls on record, while no sector has been left undamaged [13]. Contessi and De Pace [11] presented statistical evidences of instability transmission from the Chinese stock market to all other markets, especially between the end of February and the beginning of April 2020. Ozili and Arun [8] emphasized that in the week of 24 February 2020, the largest 10 companies in the United States lost important amounts of money from their accounts. Furthermore, Fernandes [12], analysing the trend of the USA stock market, showed that it has fallen below 30% from its peak during March 2020. Following the USA stock market, Fernandes [12] studied the evolution of other major economies of the world and indicated that the stock market performances of the United Kingdom, Germany, Brazil, and Columbia were even worse than the performance registered in the USA stock market, with decreases of 37%, 33%, 48%, and 47%, respectively.

Various studies reported that the evolution of new cases of coronavirus and new deaths caused by it had a significant impact on the stock market. For instance, Alber [14] conducted a panel data analysis and showed that stock market for China, France, Germany, and Spain has been influenced by new cases of COVID-19 more than by deaths in the previous period from 1 March 2020 to 10 April 2020. Other authors (e.g., [4,5,6,7,15]) corroborated that the coronavirus disease negatively influences the stock markets in several countries.

Kartal et al. [4] focused on the East Asian Countries (i.e., China, Hong Kong, Japan, Mongolia, Korea, and Taiwan) in order to study the reaction of their main stock market indices to the COVID-19 pandemic. The study covered the period between 2 January 2019 and 30 September 2020, and the authors set the two sub-periods by the date of the first recorded case of COVID-19 (i.e., the Pre-Pandemic and Pandemic Periods). The results of the quantile regression models showed the negative effect of the pandemic on the studied stock markets. Al-Awadhi et al. [5] studied the effect of the COVID-19 pandemic on the companies included into the two of the most important stock market indices in China (i.e., the Hang Seng Index and the Shanghai Stock Exchange Composite Index) over the period 10 January 2020–16 March 2020 using a panel data approach and found that they were significantly negatively related to both the daily growth in total new cases and total new deaths caused by the coronavirus. Similar results were reported by Adenomon et al. [15] in Nigeria, who used the GARCH models and found a negative effect of the pandemic variables on the stock market for the period between 2 January 2020 and 16 April 2020.

Elsayed and Abdelrhim [6] used multiple regression analysis in a study investigating the impact of new cases and deaths due to COVID-19 between 1 March 2020 and 10 May 2020 on the Egyptian stock market on 17 sectors (e.g., IT, Media, and Communication Services; Food, Beverages, and Tobacco; Banks; Trade and Distributions; Education Services; Industrial Goods, Services and Automobiles; Health Care and Pharmaceuticals; Building Materials; Travel and Leisure; Energy and Support Services). The authors reported that the returns of most sectors appear to be more sensitive to cumulative mortality indicators than to daily deaths, while the new cases from coronavirus are more important than the cumulative cases of coronavirus.

In Romania, the study of Gherghina et al. [7] showed the high impact of the COVID-19 on the Romanian stock market (i.e., BET index) between 31 December 2019 and 20 April 2020 due to the crisis in China and Italy. The results of the ARDL modelling based on the new cases in China due to COVID-19 had the most significant coefficients compared to the model containing the number of new deaths in China. As for the influence of the pandemic variables in Italy, new cases had an influence on the BET index, while the new deaths showed no significant effect.

The pandemic context has prompted unparalleled global responses, and on the national level, authorities implemented travel bans, confinement, and lockout measures with the purpose of preventing the spread of the disease [16]. In this way, the economies were significantly affected as people were asked to stay at home, the severity being felt in various sectors (e.g., tourism, hospitality, travel, sports, finance, environment, health, education), causing, ultimately, the contraction of GDP. Ozili and Arun [8] studied the impact of social distancing policies on the stock market (measured by leading stock market indicators of Japan, the United Kingdom, the United States, and South Africa) using a panel approach. The main results showed that in the period from 23 March 2020–23 April 2020, the stock market was negatively influenced by the number of lockdown days and by the international travel restrictions, but positively by restrictions on internal movement. Following Ozili and Arund [8], Chowdhury et al. [9] extended the analysis including some countries from Europe (e.g., Italy, Germany, Spain) in a similar panel approach. The authors showed that the number of lockdown days and the number of new coronavirus patients, restrictions on internal movement, and international travel restrictions affected the stock market price.

Zoungrana et al. [10] focused on the stock returns of companies listed on the West African Economic and Monetary Union’s stock market (i.e., Benin, Burkina Faso, Ivory Coast, Guinea-Bissau, Mali, Niger, Senegal, and Togo) during the COVID-19 outbreak. Their aim was to examine the effect that governments’ anti-COVID-19 measures had on companies generally and on the sectors representing the majority of the listed companies (i.e., industry, finance and distribution). At the general level, while social distancing and governance measures were associated with a positive reaction of the market, movement restrictions and lockdown measures contributed to a decline in the stock value. The results also showed that the movement restrictions had a significant and negative impact on the stocks returns of companies from the three sectors, while concerning lockdown measures, companies from the industry and finance sectors were more affected than the ones from the distribution sector. In Indonesia, Utomo and Hanggraeni [17] also analysed the stock market, focusing on several companies at both the general level and at the sector level. The results of the fixed-effects panel regression revealed that, at the general level, in addition to new cases and new deaths caused by the COVID-19 pandemic, the stock market was significantly influenced by the lockdown measures as well. Moving on to the analysis of the companies by sector, the authors showed that the ones from some sectors (e.g., basic industry; consumer goods; mining and trade, service, and investment) were more affected than the others (e.g., agriculture and infrastructure).

In addition to the movement restrictions, many central banks adopted expansionary monetary measures to stimulate the economy through interest rate adjustments [8,18,19]. The current monetary policy of the main central banks (e.g., European Central Bank, Federal Reserve) is quite expansionary as they are purchasing a significant part of the public debt issued by their national governments. As shown by Fernández et al. [18], there are strong similarities between the responses of central banks to the Great Recession and their current reactions to the COVID-19 economic crisis; specific stimuli have been encouraged for injecting liquidity into markets suffering from the negative consequences of the pandemic. Ozili and Arun [8] made a comprehensive overview of monetary policy measures implemented in the countries of Central and Eastern Europe, the Middle East, and Africa between January 2020 and April 2020. In most countries, the level of monetary policy interest rate gradually decreased by different percentage points (e.g., less than 1 pp in Russia and Israel; 1 pp in the Czech Republic and Poland; more than 1 pp in Romania, Ukraine, Egypt, Ghana, and South Africa). In case of the United States, Feldkircher et al. [19] showed that the Federal Reserve was successful in stimulating growth and its monetary policy triggered a depreciation of the USA dollar that supported the external competitiveness of the USA economy.

In the international economic context, one area that has received considerable attention refers to the impact of crude oil price volatility on the stock market. The price of crude oil represents an important barometer for economies, as changes in the price can be key indicators for countries. In addition, while the COVID-19 pandemic has triggered a sharp rise in uncertainty, the shock to the oil market and the stock market is unprecedented. Zhang and Hamori [20], analyzing the evolution of crude oil market and the stock market for the USA, Japan, and Germany between January and September 2020, found that although the crude oil and stock markets were gradually returning to normal at that period, they remained unstable as the COVID-19 pandemic continued. According to Salisu et al. [21], although the COVID-19 pandemic may produce a short-term economic impact, this shock could also adversely affect the oil price–stock nexus. Analyzing a set of 15 countries from different continents in the period from March–May 2020, the authors emphasized the fact that the probability of having negative oil and stock returns may be due uncertainty associated with the relevant markets. Taking into consideration the price–stock nexus, Zhang et al. [22] highlighted that while oil prices predicted stock returns in both the pre-COVID-19 and COVID-19 periods, the predictive effect of oil prices had declined by around 89% in the COVID-19 period compared to the pre-COVID-19 period. The main implication of their results has roots in the academic literature, which shows that investors can devise successful trading strategies by using information on oil prices. In addition to the price of crude oil, another indicator that can be used in order to measure the general tendency of the economy is the Purchasing’ Managers’ Index (PMI). Several studies from the academic literature identified significant relationships between the PMI and the stock market. For instance, the study of Gomes and Peraita [23] revealed that, in the period between 2003 and 2014, the stock market for Germany, France, Italy, and Spain were significantly affected by the PMI’s announcements, particularly during the economic and financial crisis. In a similar approach, Wang and Yang [24] stated that if positive (or negative) PMI news is announced, then investors may rebalance their portfolios by buying (or selling) stocks, thus leading to a rise (or fall) of the stock market. Accordingly, one of the main results obtained by the authors in the case of China during the January 2005–March 2008 period showed that PMI news that is positive and accords with upwards economic conditions have significant positivity effects on stock market returns.

Based on the main lines of research identified in the literature review, we have formulated the following research hypotheses:

**Hypothesis** **1 (H1).** *The BET index has been affected by the number of new cases and by new deaths due to the SARS-CoV-2 virus.*

**Hypothesis** **2** **(H2).** *The BET index is significantly influenced by measures adopted by the national authorities.*

**Hypothesis** **2a** **(H2a).** *The BET index has been negatively influenced by the social distancing measures: restrictions of internal movement and international travel control.*

**Hypothesis** **2b** **(H2b).** *The BET index has been positively influenced by the monetary policy measures: reduction of monetary policy interest rate.*

**Hypothesis** **3** **(H3).** *The BET index is significantly influenced by the international economic context.*

**Hypothesis** **3a** **(H3a).** *The BET index has been negatively influenced by the price of crude oil.*

**Hypothesis** **3b** **(H3b).** *BET index has been positively influenced by the economic activity from Europe, the USA, and China.*

**Figure 1:**
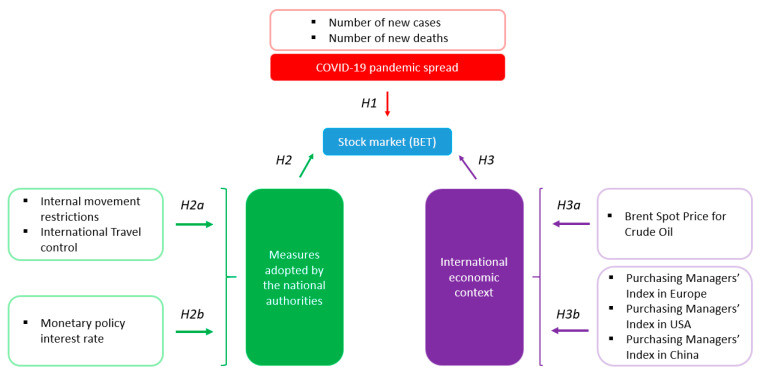
Research framework. Source: authors’ contribution.

Following the arguments mentioned above and available in the existing literature, we constructed the research framework presented in Figure 1.

## 3. Data and Methods

### 3.1. Data

To conduct this study, daily data were collected over 416 days, starting with 11 March 2020, when the authorities took first measures against the spread of the new Coronavirus in the territory of Romania, until the 30 April 2021, the date for which the latest data were available for the studied variables. To measure the impact of COVID-19 on the stock market, we considered the BET index, which is the reference index of the capital market in Romania.

The analysis of this index is highly significant for the overall state of the Romanian economy taking into account the two events that occurred during the studied period: the beginning of the health crisis and, starting with the 21 September 2020, the inclusion of the Romanian capital market into the index of emerging markets as it appears in the classification of FTSE Russell, the global supplier of indexes.

The influence variables for BET index evolution were grouped into following categories: variables related to the spread of COVID-19 pandemic, variables related to measures adopted by the authorities against COVID-19 and variables related to the international economic context. Table 1 below presents data descriptions.

### 3.2. Empirical Methodology

**Figure 2:**
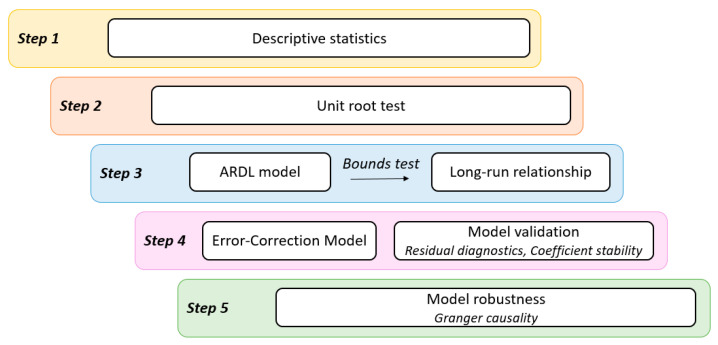
Empirical research methodology. Source: authors’ contribution.

In order to achieve the proposed objectives, we apply the following empirical research methodology (Figure 2), which comprises the following five steps: (1) analyzing the descriptive statistics; (2) testing for the unit root without and with structural breaks for the selected variables; (3) identifying the types of relationships among the variables; (4) modelling the relationships among variables; and (5) verifying the model robustness.

Data processing in the form of graphical representations and indicators of descriptive statistics represents a preliminary step in the statistical or econometric analysis as it offers relevant information for the process of verifying and cleaning the database and for the identification of necessary data transformations.

To measure the impact of the COVID-19 pandemic on stock markets, we applied the autoregressive distributed lag (ARDL) cointegration method, developed through the contributions made by Pesaran and Shin [25] and Pesaran, Shin, and Smith [26]. The use of this method is adequate for studies with a high number of variables as the ARDL cointegration method has the advantage of verifying the existence of long-run relationships between variables that have different orders of integration, while the results of the analysis are robust for an incorrect specification of the order of integration [27,28]. However, the ARDL methodology imposes that no variable should be integrated of second order or I(2) [7]. Therefore, the first phase of empirical methodology includes the analysis of stationarity of the analysed variables using the Augmented Dickey–Fuller (ADF) test [29]. As this test does not account for the potential structural breaks in the time series and could provide spurious results when the data are trend-stationary with a structural break, the Zivot–Andrews unit roots test with one structural break [30] will be also used.

The ARDL Bound test model used in this study is expressed as follows [31,32]:

(1) $$\Delta Y_{t}=\overset{p-1}{\underset{i=1}{\sum }}\beta_{i}\Delta Y_{t-i}+\overset{k}{\underset{j=1}{\sum }}\overset{q_{j}-1}{\underset{l_{j}=0}{\sum }}\delta_{j,l_{j}}\Delta X_{j,\;t-l_{j}}+\gamma Y_{t-1}+\overset{k}{\underset{j=1}{\sum }}\phi_{j}X_{j,\;t-1}+\lambda z_{t}+u_{t}$$

where $Y_{t}$ is the dependent variable of the ARDL model, which is the BET in this study; $X_{j,t},\;j=\bar{1,\;k}$ are the independent variables, NC/ND, IR, Crude_oil, PMI_EURO, PMI_USA and PMI_CHN; *k* is the number of independent variables; $z_{t}$ is an s × 1 vector of deterministic variables such as the intercept term, RIM, ITC, dummy variables, or time trends; Δ is the first difference operator; $u_{t}$ is the error term; $\beta_{i}$ and $\delta_{j,l_{j}}$ are the coefficients of the terms that indicate the short-run relationships; γ and $\phi_{j}$ are the coefficients of the terms that indicate the long-run relationships; p and $q_{j}$ represent optimal lags for the variables $Y_{t}$ and $X_{j,t}$ identified based on information criteria; and λ is the coefficient attached to a term of the vector $z_{t}$.

The Bounds test consists of testing the null hypothesis of no long-run relationship between the considered variables by using Fisher statistics. Pesaran et al. [26] calculated the critical values for F-statistics. These critical values are obtained for different circumstances and define an interval for which the lower bound is determined based on the hypothesis that all variables are I(0), while the upper bound is calculated based on the hypothesis that all variables are I(1). The critical values proposed by Pesaran et al. [26] are useful and efficient for large sample sizes. According to the decision rule, if the calculated value of the F statistic is higher than the upper bound, the null hypothesis is rejected and the variables are cointegrated; if it is inferior to the lower bound, the null hypothesis of no cointegration cannot be rejected and, therefore, no long-run relationships can exist; if it lies between the, the cointegration test is inconclusive.

According to the cointegration test results, if there are long-run relationships between the variables under consideration, an error correction model (ECM) is applied and its equation is:

(2) $$\Delta Y_{t}=\overset{p-1}{\underset{i=1}{\sum }}\beta_{i}^{\prime }\Delta Y_{t-i}+\overset{k}{\underset{j=1}{\sum }}\overset{q_{j}-1}{\underset{l_{j}=0}{\sum }}\delta_{j,l_{j}}^{\prime }\Delta X_{j,\;t-l_{j}}+\theta ECT_{t-1}+u_{t}^{\prime }$$

where *θ* is the coefficient of error correction term (ECT). This coefficient must be statistically significant, negative, and subunitary, showing the speed at which the dependent variable regains balance following the shock produced within the system. The ECT is obtained as the residual component of the equation estimated between the dependent variable *Y_t_* in level and the independent variables $X_{j,\;t},\;j=\bar{1,\;k}$ in level, i.e., the difference between the BET observed variable and the determinist component of the long-run model:

(3) $$ECT_{t-1}=Y_{t-1}-\left(\alpha_{0}-\overset{k}{\underset{j=1}{\sum }}X_{j,\;t-1}\right)$$

To validate the previously estimated model, the stability of model coefficients and the residual component are verified.

The robustness of the results obtained in the ARDL models is verified applying VECM Granger causality, which reveals information regarding the significant causal relationships between variables both in the short and long-run.

The ARDL approach for cointegration has the advantage of being less restrictive regarding the conditions of application, which facilitates the inclusion into the analysis of a sufficiently large number of variables with different orders of integration, as is the case of the present study. This methodology is suitable to achieve the objectives proposed in our research, as follows: (i) the cointegration test responds to the objective of identifying the existence and the type of relationships among the studied variables; according to the test results, it can be determined whether there are significant connections between the variables and whether they are in the long or short run; (ii) depending on the type of connections between variables, an ECM model can be developed in the case of the existence of cointegration relationships or an ARDL model on short-term or in case of lack of cointegration relationships. In this way, the objective of describing the evolution of the BET index against the analyzed influence factors is achieved. The ECM model has the advantage of measuring the impact of independent variables on the dependent variable in both long and short run, providing useful information to policy makers on effective measures that can be taken to stimulate the favorable evolution of the BET index in the context of the COVID-19 pandemic.

## 4. Results

The first part of the empirical analysis comprises a graphical and a descriptive analysis of the BET index’s evolution against the analysed variables. The second part presents the results of the application of the ARDL-Bound’s cointegration methodology, as well as the econometric modelling of the BET index in the context of the COVID-19 pandemic.

### 4.1. The Evolution and Summary Statistics of the BET Index and the Studied Influence Variables in the Context of the COVID-19 Pandemic

The existence of the COVID-19 pandemic has been officially reported to have started in December of 2019 in a province of China, spreading over all continents. Although in the beginning, the virus was present in countries around the East Asian region, as days passed, the epicentre of this virus moved to Europe and other continents [32,33]. In Romania, the first patient infected with the virus was registered on 26 February 2020, the number of the infections increased daily, reaching 20 new cases on 11 March 2020. Then, the World Health Organization declared the COVID-19 a pandemic, and the governments of different countries started adopting measures for keeping the situation under control.

**Figure 3:**
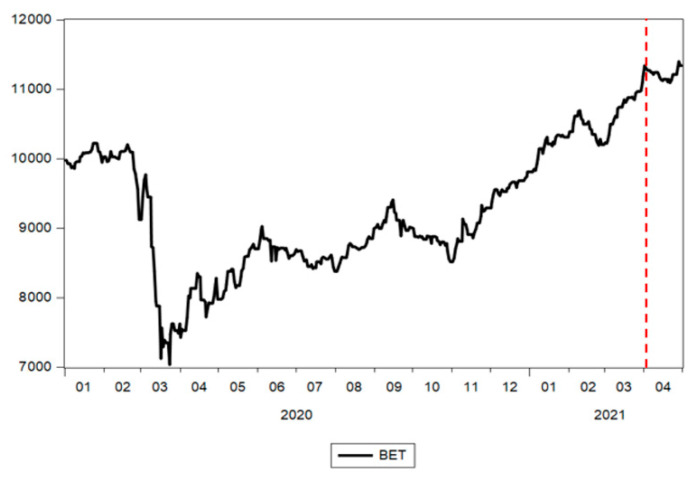
Evolution of the BET index. Source: authors’ contribution using EViews 10 software (IHS Global Inc., Irvine, CA, USA).

The year 2020 was atypical for the evolution of the local capital market, as well as for the global markets, taking into account that the COVID-19 pandemic led to one of the most severe health crises in the recent history of humankind. The first signs of the pandemic at the Bucharest Exchange appeared at the end of February, a decrease that followed the trend of the European and international markets. The period of decline was fuelled by the panic brought about by the coronavirus. These evolutions on the stock market in Romania were reflected by the evolution of the BET index between 1 January 2020 and 30 April 2021 shown in Figure 3. Similar to other stock markets, the Romanian stock market recovered slowly after the initial COVID-19 shock. In contrast to the European markets, the stock markets in the USA and China recovered significantly in the first seven months of 2020 [34].

The key event of this period occurred in September of 2020, when Romania entered into the category of secondary emerging markets that gave Romanian companies the opportunity to gain an easier access to investment funds, thus benefitting from higher confidence of investors in stock markets [35]. As a consequence, we can identify on the graph a structural break in October 2020, when it positively affected the evolution of the BET, changing the slope of the evolution trend.

**Figure 4:**
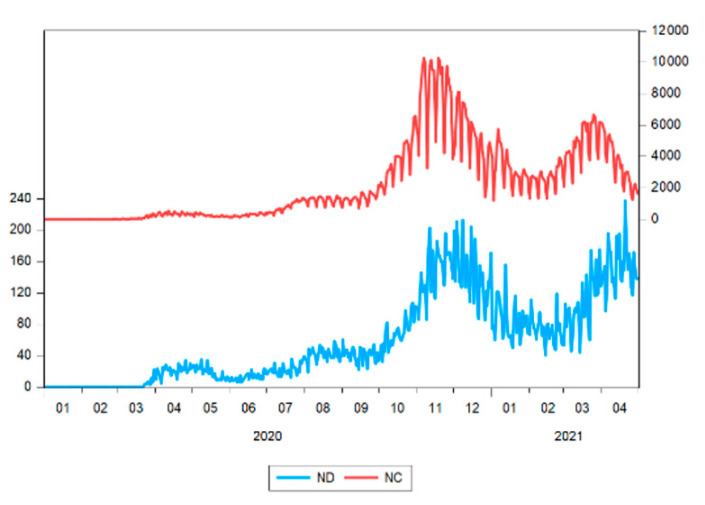
Evolution of COVID-19 pandemic in terms of the number of new deaths (ND) and number of new cases (NC). Source: authors’ contribution using EViews 10 software.

**Figure 5:**
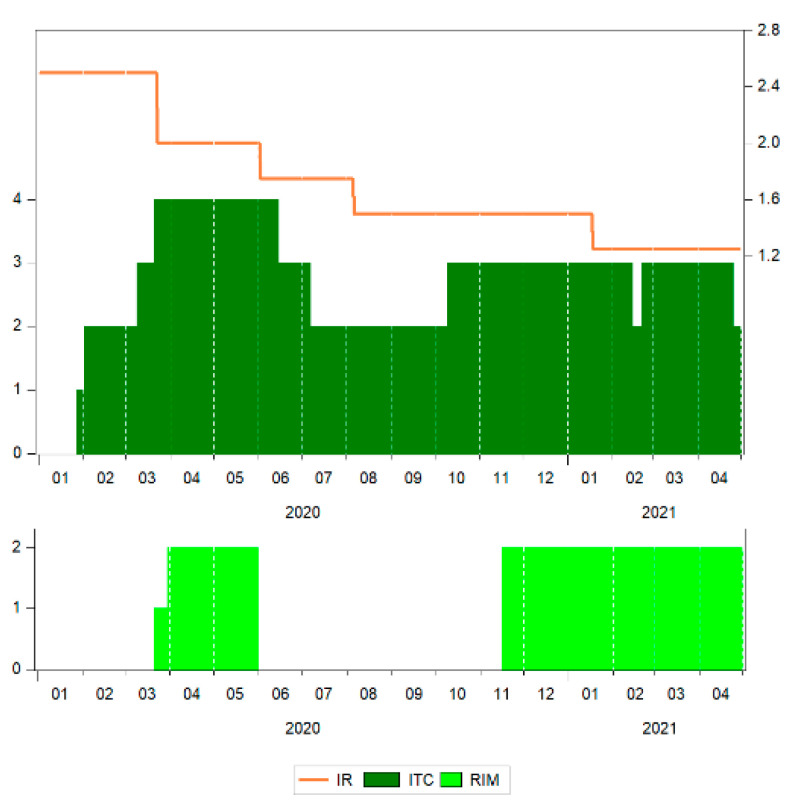
The response of national authorities in Romania in terms of the monetary policy interest rate (IR), international travel control (ITC) and internal movement restrictions (RIM). Source: authors’ contribution using EViews 10 software.

Figure 4 shows the evolution of the COVID-19 pandemic in terms of the number of new cases and new deaths, while Figure 5 shows some of the measures implemented by the national authorities in Romania over the period from 1 January 2020 to 30 April 2021.

Until October 2020, the pandemic variables in Romania showed low values compared to the rest of the period (values below 100 new deaths (ND) and below 1500 new cases (NC)). In the following period, the levels for these variables strongly increased, the incidence of the virus increasing in two waves reaching the highest values in November 2020 and March 2021, respectively. Except for January and February 2021, when the values were closer to the period before October 2020.

The rapid spread of COVID-19 has created a wide range of responses from governments, interventions having been taken to diminish the virus incidence. There could be found such common measures as international travel controls (ITC) and restrictions on internal movement (RIM). As for ITC in Romania, in April–May 2020, measures included ban for travelling in all regions, or total border closure, and between July and September of 2020, all people arriving from high-risk regions having been quarantined, during October 2020–April 2021, the ban being applied only for arrivals from some regions. In what regards RIM, there were internal movement restrictions in place in Romania for most of the period analysed, except for June–October 2020, when there were no such measures.

Another measure sustaining business operation comprised the change in the level of monetary policy interest rate (IR). In the context of the health crisis that was expected to be followed by an economic crisis, the National Bank of Romania decided to gradually decrease the interest rate, from 2.5 in March 2020 to 1.25 in April 2021, in line with the monetary policy measures adopted at the European Union level (Figure 5).

**Figure 6:**
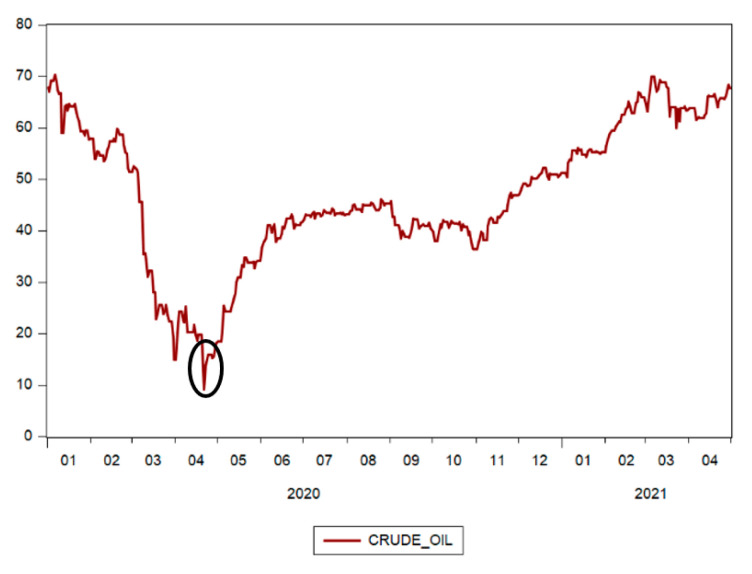
Brent Spot Price for crude oil. Source: authors’ contribution using EViews 10 software.

The evolution of the Brent price for crude oil is shown in Figure 6. Crude oil is a key determinant factor for the general state of global economy. In the context of the COVID-19 pandemic that led to a decrease in economic activity, the demand for oil also decreased. The decrease in demand contributed to the decrease in the price of crude oil at the beginning of 2020, reaching its record low on 20 April 2020 for WTI (West Texas Intermediate) and on 21 April 2020 for Brent Crude oil, respectively, each being major benchmarks for oil pricing. The price of crude oil recovered the level it had before the pandemic only in February of 2021 against the background of hopes that economic recovery could be more accelerated after the beginning of vaccination.

**Figure 7:**
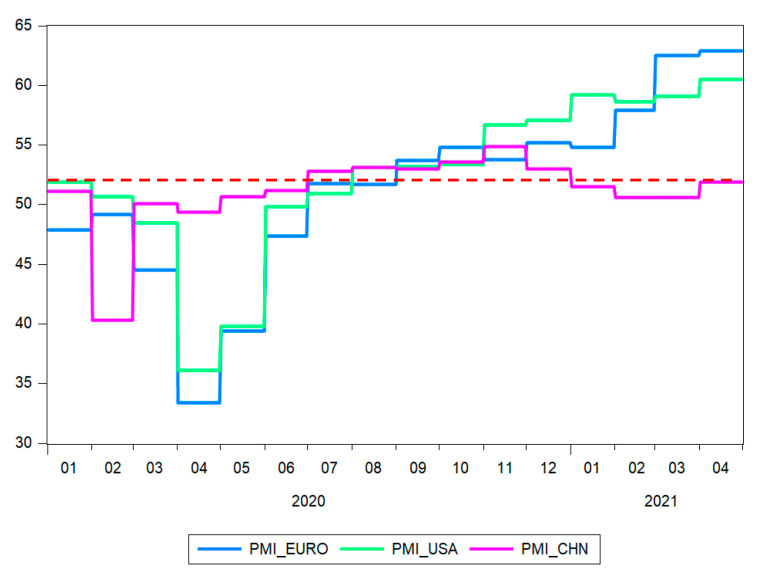
Purchasing Managers’ Index. Source: authors’ contribution using EViews 10 software.

At the beginning of 2020, the impact of COVID-19 on the manufacturing sector led to a record low on the Purchasing Managers’ Index (PMI) (Figure 7): in Europe, in April, when it reached the level of 33.4, although the previous months showed values also under 50; in the USA, the PMI reached a level similar to Europe, going under 50 in March, and the lowest level of 36.1 also in April; in China, the collapse of the manufacturing sector was not as strong as in Europe or the USA, and it reached its lowest level of 40.3 PMI in February, and then recovered quickly. The PMI grew over 50 in July of 2020 in Europe and the USA, while in China, it was over 50 during the entire period after the February decline, except for April, when there was a minor decline, without long-term consequences.

It should be noted that PMI values under 50 show a contraction of activity, and an expansion, if these are over 50, while a value of 50 showing no changes compared to the previous month [36].

The manufacturing sector had an unprecedented decrease in output in the months mentioned above as the measures taken for preventing the spread of coronavirus led to closure of unessential business, delays in supply chains and a decrease in global demand. Starting with July 2020, The PMI in Europe and the USA showed expansion values, and high leaps in manufacturing activity, as recorded in April 2021, reflected an increase in the stock market quotations.

The descriptive statistics of the variables are shown in Table 2. We analyzed the degree of variability and the representativeness of the mean for the studied series by using the coefficient of variation, calculated as a percentage between the standard deviation and the mean, $v=\frac{SD}{mean}\cdot 100$. It is found that the BET index (v=11.20%) had a low variation, and, thus, the mean of 9237.288 points, is representative for the data of this series. Regarding the Purchasing Managers’ Index, the lowest variation was in China, $v_{CHN}=2.868\%$, and in Europe and the USA, the degree of variation reached comparable value of lower intensity, $v_{EURO}=15.28\%$ and $v_{USA}=13.390\%$, respectively. The highest degree of variation was found for crude oil, with a variation of coefficient of 30.620%, showing that the mean is moderately representative for the data of this series.

The distribution of variables for Romania (BET index, the number of new cases, the number of new deaths) shows a positive asymmetry, positive deviations from the average being predominant compared to the negative ones. It is positive for the evolution of the BET index and negative for the evolution of NC and ND.

The distribution of variables for the international markets, such as crude oil for the Purchasing Managers’ Index for Europe and the USA, shows a negative asymmetry. The only exception is the PMI for China, showing that the shock produced by COVID-19 on the manufacturing sector operations was of low-intensity and short-term. The value of the coefficient of kurtosis for the BET index is lower than three, indicating a platykurtic distribution, i.e., a low level of risk for the local stock market. In addition, the Jarque-Bera test indicates that all analyzed series are not normally distributed, except for crude oil, at the 10% level of statistical significance.

Taking into consideration the large sample size and absolute values less than two for all asymmetry coefficients, we can conclude that the distributions of the variables considered in the present study do not have significant problems regarding asymmetry. At the same time, it was found that all distributions have a low, at most moderate, degree of variation. Therefore, given the nature and statistical peculiarities of the data used in this study, we considered it appropriate to use the original data in the analysis.

To establish the relationships among the variables, we have analyzed the bivariate correlation coefficients (Table 3).

The results indicate that the monetary policy interest rate is strongly negatively correlated (below −0.7) with the BET index, showing a positive effect of this measure on the monetary policy adopted by the National Bank of Romania. In addition, we observe that the BET index is strongly and positively correlated (over 0.7) with the economic operations in Europe and the USA, described by the Purchasing Managers’ Index (PMI) and is not strongly correlated with PMI in China. There is a strong and positive correlation between the economic operations in Europe and the USA, while their correlation with the economic activities in China is of moderate intensity (below 0.5). The international economic environment, described by the oil price, has a positive significant influence on the BET index.

In conclusion, the evolution of the BET index is the result of simultaneous influences of the European internal and global economic environments.

### 4.2. The ARDL Cointegration Analysis

Considering the results of the ARDL methodology of cointegration, according to which, the variables included in the analysis have a lower order than two, the first step of the analysis needs to be that of stationarity (Table 4).

The results of the ADF unit root test showed that all the variables had an integration order below two, so it is appropriate to apply the ARDL Bounds cointegration approach. We also performed the Zivot–Andrews unit root test to identify the existence of any significant structural break which could be used for stabilizing the models.

Considering the particularities we identified in the evolution of BET in our empirical analysis, we split the analysed period into two sub-periods: 11 March 2020 to 28 October 2020 and 29 October 2020 to 30 April 2021.

Furthermore, we present the empirical results of the analysis investigating the impact of the COVID-19 pandemic on the BET using the ARDL Bounds cointegration approach for the two sub-periods. The analysis of stationarity on the considered variables in the case of each sub-period confirmed the condition for the application of the ARDL-Bound cointegration approach.

For the robustness of the results, we consider two models for each sub-period: the M1a and M1b models for the first sub-period, and the M2a and M2b models for the second sub-period.

We included NC as a pandemic variable into the M1a and M2a models, and ND as a pandemic variable into the M1b and M2b models (Table 5).

The results indicated cointegration relationships between the variables in all models for a significance level of, at least, 5%. For these results, we estimated the ARDL models within an error correction model (ECM) framework to confirm the long-run relationships.

Table 6 reports the long-run coefficient estimates, the coefficient of the error correction terms (ECT_t−1_), and the diagnostic test statistics for each of the models.

In both sub-periods, pandemic variables had a negative influence on the evolution of BET. In addition, an inverse relationship between the monetary policy interest rate and the BET was identified, meaning that the decision of the National Bank of Romania to lower the rate contributed to an increasing of the BET. Regarding the impact of the international context on BET, for each sub period, the influence of crude oil was different: positive, for the period before Romania’s entering the secondary emerging markets, and negative, after that. The influences of Purchasing Managers’ Indices in Europe and China remained the same for both sub-periods (positive and negative, respectively), while, in the USA, it changed its influence from negative in the first sub-period to positive in the second one.

Table 6 indicates that the coefficients of the ECT_t−1_ terms are all significantly negative and smaller than one in absolute terms. The deviation from the long-term path of BET is corrected for each period in Romania by 19.3%, 11.8%, 37.5%, and 10.5%, respectively. In the short run (Table 7), it can be noticed that the variables used as proxy for the international context and the internal measures taken by the authorities were significant in relation to BET in some lags. The sum of the significant coefficients to the particular lags measures the cumulative effect of each independent variable on the dependent one.

The results that have been obtained from analysing the BET index in relation to the price of crude oil in the two sub-periods confirm the highlighted contradictions in the extensive research carried out by researchers and practitioners on this very complex subject. Their conclusions vary from the presence of a strong connection, which is mainly the other way around, without excluding the variant of a positive connection or the one showing the absence of the connection. Thus, in the first sub-period, when the Romanian capital market was still in the process of modernization and reformation in order to become a viable destination for investors, the M1a model indicated a negative cumulative relationship on the short run between the BET index and the price of crude oil; as the price of crude oil increases, stock valuations are driven down; as the price of crude oil decreases, stock valuations increase. Among the results obtained for the second sub-period, characterized by the fact that the Romanian capital market was included in the category of emerging markets, the M2a model highlights a positive cumulative impact on the short-run of the price of crude oil on the BET index, meaning that an increase in the price of crude oil will lead to an increase in the stock market.

The relationship between the economic context at the national or international level and stock markets is a fundamental one. It is generally proven that the stock markets reflect the results of the economic activities. The cumulative impact on the short-run of the economic activity, represented by PMI variables, on the BET index was different in direction and significance for the two sub-periods studied, as well as for the countries and regions that have been considered. Therefore, in the first sub-period, the results of the M1a model indicated that the BET index was negatively influenced by the PMI of Europe, positively by the PMI of USA, and insignificantly by the PMI of China. The situation changed in the second sub-period, when the results of the M2a model showed that, while the influence of Europe’s PMI remained negative, the influence of China’s PMI became positive and the USA’s became negative.

Regarding the cumulative effect in the short-run of the monetary policy interest rate on the BET index indicated in the M2a model, it can be observed that the National Bank’s aim to protect and stimulate the economic activity as a whole has been achieved by gradually reducing the level of this key indicator of monetary policy, especially the capital market activity.

For the analysed period, these unpredicted variations produced in all economic and social activities, with influences manifested in the short or long run, have the imprint of uncertainty created by the ongoing COVID-19 pandemic.

**Figure 8:**
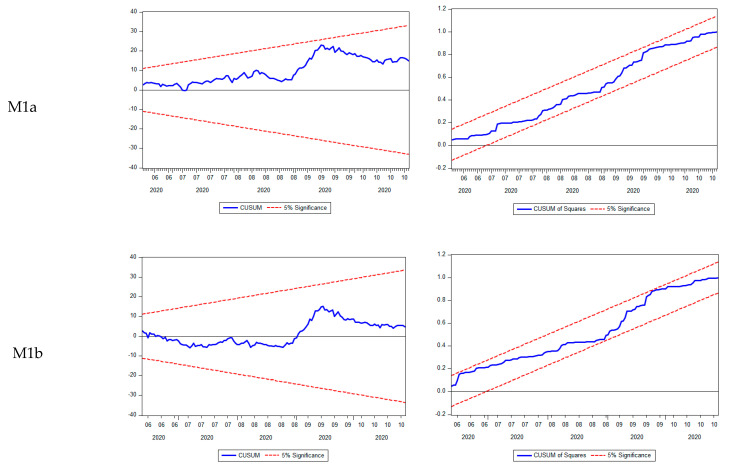

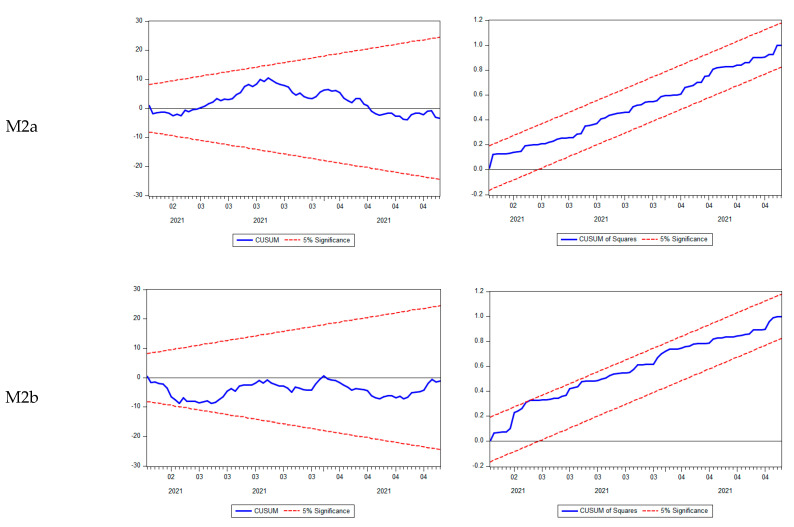
Plot of Cumulative Sum of recursive residuals (CUSUM) and Cumulative Sum of Square recursive residuals (CUSUM of Squares) tests for the parameter stability. Source: authors’ contribution using EViews 10 software.

For validating the models, we checked the hypotheses formulated on the residual component and the stability of coefficients (Table 6 and Figure 8). All models were validated, and all hypotheses formulated on residual and coefficients were verified.

The robustness of the results from the validated models was verified through the VEC Granger causality models. Granger [37] suggested that in the presence of cointegration, the VECM Granger causality framework is an appropriate approach to detect the long-and-short runs causal relationship between variables. Table 8 presents the results for the “short-run” and “long-run” Granger causalities.

In the long-run, BET is Granger caused by all the determinants considered. In the short-run, each sub-period has a particular behaviour and the independent variables have significant influences at different lags.

## 5. Conclusions

As one of the most recent and significant phenomena, the COVID-19 pandemic has affected the stock markets severely [4,11,13]. In light of this, the study focuses on determining how the pandemic has affected the Romanian stock market (measured by the BET index) over the period from 11 March 2020–30 April 2021 using three categories of variables: pandemic variables (number of new cases and new deaths due to coronavirus), variables reflecting the measures implemented by the national authorities (restrictions of internal movement, international travel control, and monetary policy measures), and variables describing the international economic context (crude oil price and Purchasing manager’ Index for Europe, the USA, and China).

The empirical analysis was conducted for the two sub-periods (i.e., 11 March 2020–28 October 2020 and 29 October 2020–30 April 2021) defined by a historical moment for the Romanian economy: its inclusion into the category of secondary emerging markets. As a result, Romanian companies can more easily access investment funds, and investors have more confidence in the capital market. This is seen in the changed trajectory of the BET’s evolution at the end of October 2020 when a structural break in the trend was identified, and it started to have a significant and positive slope. This slope described the favourable evolution of the BET index in the context of the BSE becoming an emerging market. In addition, the results showed a positive influence in the long-term of the European economic context on the BET index. This positive aspect is sustained by the existent initiatives at the level of the European Union regarding the common interests for helping Member States finance crisis response and recovery measures following pandemics [3,38].

The results of the ARDL Bounds test indicated that there are long-run relationships between BET and the studied variables. The results of the empirical analysis showed that the formulated research hypotheses have been confirmed. Therefore, the hypothesis H1 on the negative influence of the pandemic variables has been validated for both sub-periods in the long-run. Hypothesis H2 regarding the fact that the BET index is significantly influenced by measures adopted by the national authorities has been confirmed through the two sub-hypotheses defined. The hypothesis H2a, referring to the negative influence of the movement restriction policies on the BET, has also been validated but in the short-run, due to the fact that most of the sectors in the economy were affected by them. The hypothesis H2b, concerning the influence of the reduction of monetary policy interest rates on the BET index, has been confirmed, meaning that the decision made by the National Bank of Romania to stimulate the economy by gradually decreasing the interest rate from 2.5 in March 2020 to 1.25 in April 2021 reached the expected outcome. Furthermore, regarding the significant influence of international economic context on BET index, the hypothesis H3 was validated, both in the case of the influence of crude oil price (sub-hypothesis H3a) and of the Purchasing Managers’ Index (sub-hypothesis H3b).

Our results are in line with the other results reported by other studies. On the one hand, we found a negative influence of the pandemic variables (e.g., [4,5,6,7]) and of the restrictions related to movement (e.g., [8,9,10]) on the Romanian stock market, and, on the other hand, we found positive influence of the monetary policy interest rate on the Romanian stock market (e.g., [18,19]).

Our contributions to the literature in the field include insight into the impact of the COVID-19 pandemic on the Romanian stock market in the long-term, covering a period of 15 months, and a methodological approach (i.e., ARDL Bound cointegration) that, to our knowledge, has been applied in few studies regarding this issue. In addition, the results could be used as a guide for national authorities in the efficient management of measures adopted against the spread of the COVID-19 disease.

The main limitation of the study lies in the fact that it is focused only on the analysis of the Romanian context. It might be helpful to extend the literature to include other European countries affected by the COVID-19 pandemic and to make a comparative analysis of the ways in which each country responded to this challenging problem. In addition, considering various numbers of countries and other statistical or econometric methods could be applied (e.g., dynamic panel modelling). Another limitation of the study refers to the fact that important economic variables were not taken into consideration (e.g., inflation, economic policy uncertainty, fiscal measures), and this study took as pandemic variables only new cases and new deaths due to the coronavirus. In future studies, other variables such as the number of tests or the number of recovered patients could produce more precise results.

## Figures and Tables

**Table 1:** Variables’ descriptions.

Symbol	Description	Source
Variable Concerning Stock Market		
BET	Bucharest Exchange Trading (BET) index is the reference index for the Romanian capital market. BET shows the evolution of the 17 most traded companies on the market regulated by the BSE, excluding financial investment companies.	Investing.com
Variables Related to the COVID-19 Pandemic Spread		
NC	The number of new cases (NC) due to COVID-19 in Romania.	Our World in Data
ND	The number of new deaths (ND) due to COVID-19 in Romania.	Our World in Data
Variables Related to Measures Adopted by the Authorities		
RIM	Internal movement restrictions (RIM)—ordinal variable with the following categories: 0—no measures; 1—recommendation not to travel between regions/cities; 2—internal movement restrictions in place.	Oxford COVID-19 Government Response Tracker (Ox CGRT) database
ITC	International Travel control (ITC)—ordinal variable with the following categories: 0—no measures; 1—Screening; 2—Quarantine arrivals from high-risk regions; 3—Ban on arrivals from some regions; 4—Ban on all regions or total border closure.	Oxford COVID-19 Government Response Tracker (Ox CGRT) database
IR	The monetary policy interest rate (IR)—a monetary policy instrument whose level was reduced by the NBR in several phases in the context of health crisis in order to reduce its impact on population and companies of Romania.	National Bank of Romania
Variables Related to the International Economic Context		
PMI_c	Purchasing Managers’ Index (PMI), where “_c” is the region or country included in the analysis, namely: EURO—Europe (PMI_EURO); USA—the United States of America (PMI_USA); CHN—China (PMI_CHN). The PMI is an indicator of economic health for the manufacturing and services sector. It is used as a proxy for the level of general economic/business activities. In the present study, we use PMI for the manufacturing sector because it includes a large number of economic variables: output, new orders, new export orders, backlogs of work, output prices, input prices, suppliers’ delivery times, stocks of finished goods, quantity of purchases, stocks of purchases, employment, future output.	The Global Economy.com
Crude_oil	Brent Spot Price for Crude Oil (Dollars per Barrel). The price of crude oil is an important index for world economy. Brent Crude is the international benchmark for oil pricing, covering approximately two-thirds of all oil pricing. Fluctuations in oil quotations are always followed by economic and social events.	Energy Information Administration

**Table 2:** Descriptive statistics of the variables.

Variable	Mean	Median	Min	Max	SD	Skewness	Kurtosis	Jarque-Bera	Prob
BET	9237.288	8882.500	7039.000	11,398.000	1034.763	0.428	2.287	21.515	0.000
NC	2536.635	1610.000	4.000	10,269.000	2458.588	1.159	3.757	103.131	0.000
ND	67.569	51.000	0.000	237.000	54.174	0.799	2.622	46.782	0.000
IR	1.591	1.500	1.250	2.500	0.297	0.903	3.570	62.304	0.000
CRUDE_OIL	44.843	43.270	9.120	69.950	13.731	–0.160	2.539	5.471	0.064
PMI_EURO	51.845	53.800	33.400	62.900	7.923	−0.832	3.176	48.564	0.000
PMI_USA	52.641	53.400	36.100	60.500	7.051	−1.110	3.301	87.118	0.000
PMI_CHN	51.935	51.900	49.400	54.900	1.490	0.170	2.179	13.689	0.001

**Table 3:** Bivariate correlations between considered variables.

Variable	BET	NC	ND	IR	CRUDE_OIL	PMI_EURO	PMI_USA	PMI_CHN
BET	1 ^(1)^							
NC	0.458 *** (10.508)	1						
ND	0.661 *** (17.935)	0.829 *** (30.252)	1					
IR	–0.852 *** (−33.202)	−0.551 *** (−13.464)	−0.648 *** (–17.323)	1				
CRUDE_OIL	0.922 *** (48.691)	0.453 *** (10.348)	0.594 *** (15.040)	–0.855 *** (–33.618)	1			
PMI_EURO	0.821 *** (29.286)	0.560 *** (13.778)	0.646 *** (17.242)	–0.862 *** (–34.628)	0.909 *** (44.509)	1		
PMI_USA	0.781 *** (25.494)	0.596 *** (15.140)	0.665 *** (18.151)	–0.831 *** (–30.419)	0.878 *** (37.357)	0.960 *** (70.535)	1	
PMI_CHN	0.017 (0.349)	0.504 *** (11.890)	0.406 *** (9.064)	–0.344 *** (–7.477)	0.150 *** (3.098)	0.414 *** (9.266)	0.440 *** (9.975)	1

Notes: ^(1)^ Value in () parentheses is t-statistic for the Pearson coefficient; *** shows the statistical significance of the Pearson coefficient at the 1% level.

**Table 4:** Results of the Augmented Dickey–Fuller (ADF) unit root test.

Variable	Trend and Intercept			Intercept		None	Order of Integration
	ϕ	Constant	Trend ^(1)^	ϕ	Constant	ϕ	
Levels							
BET	–3.160 *	3.128 ***	3.376 ***	–3.165 **	–0.236	−3.167 ***	I(0)
IR	–4.006 ***	–3.723 ***	–3.083 ***	–4.007 ***	–0.482	−4.009 ***	I(0)
CRUDE_OIL	–2.865	2.554 **	2.966 ***	–2.867 *	−0.402	−2.870 ***	I(0)
PMI_EURO	–2.615	2.612 ***	2.517 **	–2.617 *	−0.367	−2.620 ***	I(0)
PMI_USA	–2.832	2.799 ***	2.665 ***	–2.834 *	−0.544	−2.837 ***	I(0)
PMI_CHN	–1.454	1.505	–0.459	–1.609	1.621	0.410	I(1)
NC	–1.529	0.707	0.407	–1.733	1.395	−1.040	I(1)
ND	–1.931	0.450	1.461	–1.271	1.649	0.056	I(1)
First difference							
PMI_CHN	-	-	-	-	-	−20.322 ***	I(0)
NC	-	-	-	-	-	−5.514 ***	I(0)
ND	-	-	-	-	-	−5.567 ***	I(0)

Notes: ^(1)^ In the case of series with a significant linear trend, the series was detrended and the results of the ADF test for the models with intercept and none correspond to these detrended series. *, ** and *** show the statistical significance of the regression coefficient at the 10%, 5%, and 1% level, respectively.

**Table 5:** Estimated ARDL models and Bounds F-test for cointegration.

Model ^(1)^	ARDL	F-Statistic
11 March 2020–28 October 2020		
*M1a (BET | NC, IR, CRUDE_OIL,* *PMI_EURO, PMI_USA, PMI_CHN)*	(9,1,10,9,10,4,0)	3.840 **
*M1b (BET | ND, IR, CRUDE_OIL,* *PMI_EURO, PMI_USA, PMI_CHN)*	(9,1,4,2,0,9,0)	3.372 **
29 October 2020–30 April 2021		
*M2a (BET | NC, IR, CRUDE_OIL,* *PMI_EURO, PMI_USA, PMI_CHN)*	(12,8,4,9,10,5,11)	6.874 ***
*M2b (BET | ND, IR, CRUDE_OIL,* *PMI_EURO, PMI_USA, PMI_CHN)*	(1,0,0,1,5,0,1)	3.332 **
**Significance**	**Critical values**	
	**I(0)**	**I(1)**
10%	1.99	2.94
5%	2.27	3.28
1%	2.88	3.99

Notes: ^(1)^ For all models were considered exogenous categorical variables RIM and ITC. **, and *** show the statistical significance of the regression coefficient at the 5%, and 1% level, respectively.

**Table 6:** Estimated long-run coefficients for the ARDL models.

	M1a	M1b	M2a	M2b
Regressors	Coefficient (Std. Error)	Coefficient (Std. Error)	Coefficient (Std. Error)	Coefficient (Std. Error)
NC	−0.196 *** (0.066)	-	−0.017 (0.015)	-
ND	-	−8.130 (5.472)	-	2.121 (1.317)
IR	−2712.069 *** (336.361)	−3090.790 *** (624.677)	−457.481 ** (221.938)	−108.019 (656.112)
CRUDE_OIL	30.618 *** (9.876)	44.730 *** (14.625)	−54.215 *** (7.302)	−46.518 ** (20.401)
PMI_EURO	123.934 *** (39.884)	78.461 (67.276)	50.027 ** (23.723)	89.277 * (45.769)
PMI_USA	−78.584 ** (39.797)	−45.479 (57.944)	43.838 (31.908)	194.051 ** (87.706)
PMI_CHN	−205.157 (137.878)	−277.886 (222.626)	−188.809 *** (23.479)	−225.418 *** (72.373)
Constant	19,998.35 *** (6630.694)	24,892.53 ** (10,425.75)	16,674.38 *** (2726.101)	7427.581 (7094.721)
ECT_t−1_	−0.193 *** (0.034)	−0.118 *** (0.022)	−0.375 *** (0.048)	−0.105 *** (0.020)
*Diagnostic tests*				
R-Squared	0.982	0.979	0.997	0.996
Adj R-Squared	0.976	0.975	0.996	0.995
F-statistic	192.492 [0.000] ^(1)^	263.237 [0.000]	694.294 [0.000]	2329.486 [0.000]
BG	2.394 [0.094]	0.097 [0.906]	1.751 [0.178]	1.258 [0.286]
BPG	1.014 [0.458]	1.313 [0.130]	0.782 [0.860]	1.339 [0.170]
ARCH	0.745 [0.388]	0.001 [0.984]	5.174 [0.024]	1.991 [0.160]
RESET	0.021 [0.883]	0.108 [0.742]	0.001 [0.977]	2.311 [0.130]

Notes: ^(1)^ Values in [] parentheses are *p*-values for the applied tests. *, **, and *** show the statistical significance of the regression coefficient at the 10%, 5%, and 1% level, respectively. Abbreviations: ECT = error correction term; BG = Breusch-Godfrey serial correlation LM test; BPG = Breusch-Pagan-Godfrey test of heteroscedasticity; ARCH = ARCH test of heteroscedasticity; RESET = Ramsey Regression Equation Specification Error Test (RESET).

**Table 7:** Significant coefficients for particular lags in the ARDL models.

	M1a	M1b	M2a	M2b
Regressors	Lag ^(1)^ (Sum of Significant Coefficients)	Lag (Sum of Significant Coefficients)	Lag (Sum of Significant Coefficients)	Lag (Sum of Significant Coefficients)
BET	4;6;8 (−0.093)	4;6;8 (−0.279)	1;2;3;4;5;6;7;9;10;11 (2.154)	-
NC	-	-	1;2;3;4;5;6 (0.143)	-
ND	-	-	-	-
IR	3;5;6;9 (1410.384)	3 (228.093)	3 (−469.555)	-
CRUDE_OIL	0;1;7;8 (−4.267)	0;1 (15.541)	1;2;3;4;5;6;8 (77.84)	-
PMI_EURO	1;7;9 (−24.222)	-	1;2;4;9 (−71.364)	0;4 (37.162)
PMI_USA	1;2;3 (92.104)	1;2;6;8 (5.551)	4 (−44.757)	-
PMI_CHN	-	-	1;2;4;10 (175.077)	-
RIM	0 (69.103)	0 (66.831)	0 (7.374)	0 (27.050)
ITC	0 (51.092)	0 (11.023)	-	0 (−8.974)
Trend ^(2)^	-	-	0 (7.482)	0 (0.451)

Note: ^(1)^ Lags of the independent variables significantly influencing BET index. ^(2)^ For models M2a and M2b, giving the evolution of BET (Figure 3), we included a linear trend.

**Table 8:** Granger causality test results.

	M1a	M1b	M2a	M2b
Long Run	Coeff. (Std. Err.)	Coeff. (Std. Err.)	Coeff. (Std. Err.)	Coeff. (Std. Err.)
ECT_t−1_	−0.069 * (0.037)	−0.062 * (0.034)	−0.206 *** (0.074)	0.008 ** (0.003)
Short run	Lag ^(1)^	Lag	Lag	Lag
BET	4;6;8	4;6;8	1;4	-
NC	10	-	2;4;5;6	-
ND	-	-	-	1;2
IR	9;10	1;3;9	7	-
CRUDE_OIL	5;6;8	1;5;6	5;10	4
PMI_EURO	6;7	7	1;5;8;9;11	-
PMI_USA	6	6	-	-
PMI_CHN	-	-	-	-
RIM	0	0	0	-
ITC	-	-	-	-

Notes: ^(1)^ Lags of the independent variables Granger causing BET index. *, ** and *** show the statistical significance of the ECT coefficient at the 10%, 5%, and 1% level, respectively.

## Data Availability

The data that support the findings of this study are available from the corresponding author, upon reasonable request.
